# A memory-reframing intervention to reduce pain in youth undergoing major surgery: Pilot randomized controlled trial of feasibility and acceptability

**DOI:** 10.1080/24740527.2022.2058919

**Published:** 2022-06-09

**Authors:** Maria Pavlova, Tatiana Lund, Jenny Sun, Joel Katz, Mary Brindle, Melanie Noel

**Affiliations:** a Department of Psychology, University of Calgary, Calgary, Alberta, Canada; b Icahn School of Medicine at Mount Sinai, New York, New York, USA; c Department of Psychology, York University, Toronto, Ontario, Canada; d Department of Pediatric Surgery, Alberta Children’s Hospital, Calgary, Alberta, Canada; e Alberta Children’s Hospital Research Institute, Calgary, Alberta, Canada; f Hotchkiss Brain Institute, Calgary, Canada; g Owerko Centre, Calgary, Canada; h Mathison Centre for Mental Health Research & Education, Calgary, Alberta, Canada

**Keywords:** chronic postsurgical pain, adolescents, parents, memory reframing, psychosocial intervention

## Abstract

**Background:**

Three to 22% of youth undergoing surgery develop chronic postsurgical pain (CPSP). Negative biases in pain memories (i.e., recalling higher levels of pain as compared to initial reports) are a risk factor for CPSP development. Children’s memories for pain are modifiable. Existing memory-reframing interventions reduced negatively biased memories associated with procedural pain and pain after minor surgery. However, not one study has tested the feasibility and acceptability of the memory-reframing intervention in youth undergoing major surgery.

**Aims:**

The current pilot randomized clinical trial (RCT; NCT03110367; clinicaltrials.gov) examined the feasibility and acceptability of, as well as adherence to, a memory reframing intervention.

**Methods:**

Youth undergoing a major surgery reported their baseline and postsurgery pain levels. Four weeks postsurgery, youth and one of their parents were randomized to receive control or memory-reframing instructions. Following the instructions, parents and youth reminisced about the surgery either as they normally would (control) or using the memory-reframing strategies (intervention). Six weeks postsurgery, youth completed a pain memory interview; parents reported intervention acceptability. Four months postsurgery, youth reported their pain.

**Results:**

Seventeen youth (76% girls, *M_age_ *= 14.1 years) completed the study. The intervention was feasible and acceptable. Parents, but not youth, adhered to the intervention principles. The effect sizes of the intervention on youth pain memories (*η_p_*^2^ = 0.22) and pain outcomes (*η_p_*^2^ = 0.23) were used to inform a larger RCT sample size.

**Conclusions:**

Memory reframing is a promising avenue in pediatric pain research. Larger RCTs are needed to determine intervention efficacy to improve pain outcomes.

## RÉSUMÉ

**Contexte:** Trois à 22 % des jeunes qui subissent une chirurgie développent une douleur post-chirurgicale chronique. Les biais négatifs dans les souvenirs de douleur (c.-à-d., se rappeler de niveaux de douleur plus élevés comparativement aux niveaux initialement rapportés) sont un facteur de risque pour le développement de la douleur post-chirurgicale chronique. Les souvenirs qu’ont les jeunes de la douleur sont modifiables. Les interventions de recadrage des souvenirs existantes ont réduit les souvenirs polarisés négativement qui sont associés à la douleur procédurale et à la douleur aprés une intervention chirurgicale mineure. Cependant, aucune étude n'a testé la faisabilité et l'acceptabilité de l'intervention de recadrage des souvenirs chez les jeunes subissant une intervention chirurgicale majeure.

**Buts:** L'essai clinique randomisé pilote actuel (RCT; NCT03110367; clinicaltrials.gov) a examiné la faisabilité et l'acceptabilité d'une intervention de recadrage des souvenirs, ainsi que l’observance de celle-ci.

**Méthodes:** Des jeunes subissant une intervention chirurgicale majeure ont rapporté leur niveau de douleur initial et post-chirurgical. Quatre semaines aprés la chirurgie, les jeunes et l'un de leurs parents ont été randomisés pour recevoir des instructions de contrôle ou de recadrage des souvenirs. En suivant les instructions, les parents et les jeunes se sont rappelé la chirurgie comme ils le feraient normalement (contrôle) ou en utilisant les stratégies de recadrage des souvenirs (intervention). Six semaines aprés la chirurgie, les jeunes ont été interviewés sur leurs souvenirs de la douleur; les parents ont rapporté l’acceptabilité de l'intervention. Quatre mois aprés la chirurgie, les jeunes ont rapporté leur douleur.

**Résultats:** Dix-sept jeunes (76 % de filles, Mâge = 14,1 ans) ont terminé l'étude. L’intervention s’est révélée faisable et acceptable. Les parents, mais pas les jeunes, ont observé les principes de l’intervention. L'ampleur des effets de l'intervention sur les souvenirs de douleur des jeunes (ηp2 = 0,22) et les résultats de douleur (ηp2 = 0,23) ont été utilisés pour déterminer une taille d'échantillon d’essai contrôlé randomisé plus grande.

**Conclusions:** Le recadrage de la mémoire est une voie prometteuse dans la recherche sur la douleur pédiatrique. Des essais contrôlés randomisés de plus grande taille sont nécessaires pour déterminer l'efficacité de l'intervention pour améliorer les résultats de la douleur.

Postsurgical pain in youth is common, often inadequately managed, distressing, and, for 3% to 22% of youth, may become chronic.^1–3^ Chronic postsurgical pain (CPSP; i.e., pain that persists for 3 months or longer after surgery and impacts health-related quality of life) contributes to the rising prevalence of pediatric chronic pain, which has been coined a “modern public health disaster.”^4,p466^ Pediatric CPSP is associated with sleep disturbances,^5^ activity limitations,^6^ and functional disability.^7,8^

According to a conceptual model proposed by Rabbits and colleagues,^9^ transition of acute to chronic pediatric postsurgical pain is influenced by demographic (e.g., age, sex), biological (e.g., genetic profile, inflammatory response), psychological (e.g., emotions, cognitions, behaviors), and social (e.g., parent, family) factors. Due to their modifiable nature and robust associations with outcomes, psychosocial factors are of particular interest and importance. Youth with high levels of general and pain-related anxiety,^8,10^ shorter presurgery sleep duration and worse sleep quality,^11,12^ and general psychosocial distress (i.e., a combination of high pain catastrophizing, pain interference, depression, and fatigue)^13^ are at greater risk of developing CPSP. Another risk factor for CPSP may involve negatively biased memories for pain (i.e., recalling higher pain as compared to the initial report). In two cohorts of youth undergoing major surgery, higher postsurgical pain intensity ratings were associated with negatively biased memories for pain 5 to 12 months later.^14,15^ Further, higher levels of baseline anxiety sensitivity and catastrophic thinking about pain during the first 24 to 48 hours postsurgery contributed to more negatively biased pain memories one year after surgery.^15^

Children’s memories are highly modifiable^16^ and can be altered by the simple act of talking about past pain experiences.^17^ However, the few existing psychosocial interventions aimed to prevent pediatric CPSP focus on pain in the short term and address modifiable psychological and behavioral factors (e.g., anxiety, psychological arousal, catastrophic cognitions),^18,19^ but pain memories have not been targeted in the context of major pediatric surgery despite their potential importance for subsequent pain experience.^20^

The existing memory-reframing interventions have been tested in the context of procedural pain (e.g., lumbar puncture, vaccine injection, dental injection)^21–23^ and have resulted in reduced negative biases in children’s memories for pain.^24^ A recent randomized controlled trial (RCT) tested the efficacy of a parent-led memory-reframing intervention in a cohort of young children undergoing a tonsillectomy.^17^ Parents learned three key principles of optimal reminiscing about past postsurgical pain, including (1) highlighting the positive aspects of the past painful experience and avoiding using pain-related words, (2) correcting negative exaggerations in pain memories, and (3) enhancing children’s pain-related self-efficacy by talking about coping strategies.^17^ Parents then used the intervention principles to reminisce with their children about the tonsillectomy. Children in the intervention group recalled their postsurgical pain in a less negatively biased way compared to children in the control group.^17^

The existing research on memory reframing is limited to procedural pain and pain associated with a minor outpatient surgery, as well as samples of young children (i.e., participants aged 4 to 9 years except for Chen and colleagues’^23^ sample of youth aged 3 to 18 years with cancer undergoing needle procedures). The feasibility and acceptability of a memory-reframing intervention, as well as its effect size on pain outcomes, in the context of major surgery with older children is unknown. The present pilot RCT aimed to fill this gap by testing the adherence to, as well as feasibility and acceptability of, a modified version of the previously used^17^ memory-reframing intervention in a sample of youth undergoing spinal fusion or pectus repair. Based on previous research,^17^ we hypothesized that the intervention would be feasible and acceptable. We hypothesized that parent–child reminiscing in the intervention group would be more intervention congruent compared to the control group (i.e., parents and children would more frequently use positive emotion-, coping-, and bravery-related words and less frequently use negative emotion-, pain-, and fear-related words). A secondary aim of the study was to calculate the observed effect size of the intervention on youth memory biases and pain outcomes to determine the sample size for a future definitive trial.

## Materials and Methods

### Trial Design

**Figure 1.**
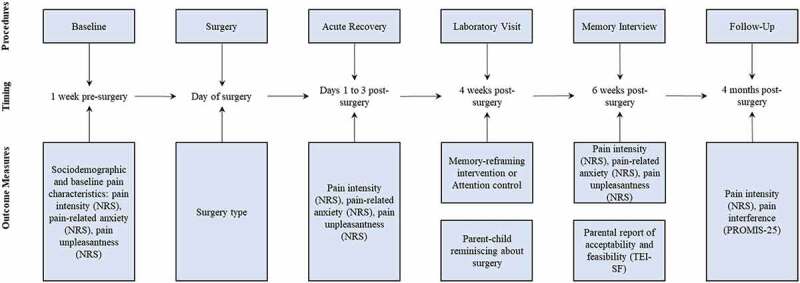
Study procedures, timing, and measures. NRS = numeric rating scale; TEI-DF = Treatment Evaluation Inventory–Short Form. For a full list of measures collected during the study, please see https://clinicaltrials.gov/ct2/show/NCT03110367.

This pilot study is a part of a larger preregistered randomized controlled trial (NCT03110367; clinicaltrials.gov, posted on April 12, 2017). The trial had a parallel group assignment with a 1:1 allocation ratio and blinded assessment of outcomes. Participants were recruited from January 2018 to June 2019. The recruitment was stopped due to insufficient funding (see Protocol Deviations section). Parent–child dyads were recruited at the Alberta Children’s Hospital. The recruitment pool was generated as follows: (1) clinic staff identified the patients scheduled for pectus repair/spinal fusion surgeries, (2) upon booking of the preop clinic visit, the administrative clinic staff obtained permission to contact from parents and share their contact details for research purposes, and (3) the study staff contacted eligible families to screen potential participants and obtain verbal consent/assent. Data were collected using a study protocol (Figure 1). Eligible families were sent and completed consent/assent forms and baseline questionnaires using secure online survey software (i.e., REDCap) approximately 1 week prior to surgery.^25^ The baseline questionnaires included measures of pain characteristics as well as multiple measures of youth functioning. For a full list of measures, please see the published trial protocol (https://clinicaltrials.gov/ct2/show/NCT03110367).

On the day of the surgery and during the acute postsurgical recovery period (i.e., typically the first 1 to 3 days postsurgery), youth reported their pain characteristics (Figure 1). Four weeks postsurgery, youth and a participating parent came to the hospital for a laboratory visit. During the visit, group allocation was revealed to the interventionist (see Randomization and Blinding section), and participants received either intervention or attention control instructions (see Interventions section for more details). The same researcher, a clinical psychology graduate student (M.P.), provided the intervention and attention control instructions. Following the instructions, parents and youth completed a reminiscing task^17^ during which they talked together about the youth’s recent surgery and postsurgical experience (i.e., the first few days after the surgery) either as they normally would (attention control group) or using the memory-reframing intervention principles (intervention group). There was no time limit. Parent–child reminiscing narratives were video- and audio-recorded, transcribed verbatim, and coded by two blinded coders for intervention adherence using an adapted coding scheme (see Intervention Adherence section for more information).

Two weeks after the laboratory visit (i.e., 6 weeks postsurgery), participants completed an established^20^ telephone memory interview to assess youth memories for pain. Memory interviews were conducted by trained research assistants who were blinded to the intervention status. The same pain measures were used for baseline assessments and memory interviews (i.e., youth reported their memories for pain and baseline/postsurgery pain using the same scales). At the end of the memory interview, the interviewer opened a sealed envelope containing the participant’s group allocation to debrief participants appropriately. Parents in the intervention group reported the intervention acceptability. Finally, 4 months postsurgery, youth reported their pain characteristics using online surveys. Participants allocated to the attention control group received a handout summarizing the intervention principles.

### Protocol Deviations

The study was registered as a randomized clinical trial (*n* = 90 parent–child dyads). However, due to insufficient funding, the trial was stopped. The primary aims were modified to assess the intervention feasibility and acceptability. The trial measures remained the same. Twenty-three dyads were recruited, with the last dyad to receive intervention/control group allocation joining the study in June 2019. Twenty-five parent–child dyads were enrolled in the study; however, due to the lack of funding, the last two dyads were not randomized to receive control/intervention instructions. Thus, the registered trial criteria were not met. Instead of intervention efficacy, the collected data were used to assess the intervention feasibility, acceptability, and adherence.

The following protocol change occurred after the study began: Instead of watching the *Planet Earth* video, in line with previous research utilizing active attention control instructions,^17^ participants in the control group received information about volunteering at Alberta Children’s Hospital. Additionally, we would like to acknowledge a mistake regarding the number of groups (i.e., three) in the registered protocol; the study had two groups. Attention control and normal reminiscing comprise one group (i.e., control group); participants randomized to the control group received attention control instructions and reminisced as they normally would about their past surgery.

### Randomization and Blinding

A researcher not otherwise involved in the clinical trial or in the delivery of clinical care performed block randomization (1:1) using a random number generator.^26^ A different researcher blinded to the study hypotheses sealed group allocations into opaque, sequentially numbered envelopes. The interventionist and other investigators were blind to group allocation. At the start of the lab visit, the interventionist (the first author, M.P.) opened the envelope with a number corresponding to the participant number to reveal group allocation. The interventionist then delivered the instructions according to the group allocation. Other investigators remained blind to group allocation until the end of the memory interview; group allocation was revealed to the memory interviewer to debrief participants appropriately and assess acceptability for those in intervention group. Statistical analyses were performed by the first author (M.P.). The analyses took place after data collection; therefore, the first author, who delivered the intervention, was not blind to group allocation at the time of data analyses; group allocation variable was labeled as “Intervention” or “Control.”

### Participants

Seventeen youth aged 10 to 18 years and one of their parents were recruited from the General Surgery and Orthopedic Surgery Clinics at Alberta Children’s Hospital. Youth were eligible to participate if they were between 10 and 18 years old and scheduled to undergo a spinal fusion or pectus repair surgery. Youth were excluded if they had severe cognitive impairment or developmental disorders, were not able to access the Internet, had serious chronic health and/or life-threatening conditions (i.e., American Society of Anesthesiologists ≥III physical status), could not speak English, and/or did not have a parent who could speak English.

### Ethics

The University of Calgary conjoint health research ethics board approved the study (REB17-0426). Participants received standard pre- and postsurgical pain management. Surgical teams were blinded to the group allocation and followed standard anesthesia and surgery protocols. No adverse effects were reported.

### Interventions

#### Control Group

Similar to previous research,^17,27^ active attention control instructions were provided. Previous research taught parents the principles of child-directed play^17,27^; however, given the older age of the current study’s participants, different information was offered. Specifically, youth and parents randomized to the control group learned about and received a handout summarizing volunteering opportunities at the Alberta Children’s Hospital. On average, the control instructions lasted 12.8 minutes (SD 4.0). During the control instructions, no information about the surgical experience was mentioned or elicited.

#### Intervention Group

Youth and parents in the intervention group learned about optimal ways of reminiscing about past experiences involving pain. This standardized intervention was previously tested in a sample of children aged 4 to 7 years undergoing tonsillectomy. Based on efficacious^24^ memory-reframing interventions for needle procedures^21–23^ and observational data demonstrating the influence of parent–child reminiscing on children’s memories for pain,^28^ the present intervention focused on three key principles of pain memory reframing: (1) highlighting the positive aspects of past surgery experience while avoiding pain-related words (e.g., *hurt, sore, pain*), (2) identifying and correcting any exaggerated memories for pain, (3) validating youth bravery during the pain experience and discussing effective pain coping strategies; to adapt the intervention for the older age group, self-validation (e.g., saying “I was brave”) was taught. In line with previous research,^17^ participants were given a rationale for the importance of pain memories (i.e., being powerful predictors of future pain) and their malleability through reminiscing and received a handout summarizing the intervention to use while talking about the surgery. Intervention instructions lasted, on average, 18.5 (SD 4.0) minutes.

Previous memory-reframing interventions^21–23^ were delivered directly to young children by researchers; the principles of the parent-led memory-reframing intervention^17^ were taught to parents to use with their young children when reminiscing. Due to the older age and cognitive capacity of the participants in the current study, we decided to teach the intervention principles to *both* youth and parents.

### Patient Engagement

The study team interpreting the results included a patient partner (J.S.) in addition to pain researchers (M.N., J.K.), a pediatric surgeon (M.B.), and clinical psychology trainees (M.P., T.L.). The patient partner provided her feedback regarding the intervention (see Discussion) and was compensated to reflect her contribution, in line with best practices.^29^

### Measures

#### Demographic Characteristics

Parents reported their age, gender, ethnicity/race, education level, and household income, as well as their child’s age, gender, and ethnicity/race.

#### Primary Outcomes

##### Intervention Feasibility

In line with previous research,^17,30^ intervention feasibility was assessed using recruitment statistics and parent report of how motivated they were to learn and understand the intervention.

##### Intervention Acceptability

The Treatment Evaluation Inventory–Short Form^31^ was used to assess the intervention acceptability. The measure is reliable and valid.^31^ Parents also reported whether they used the intervention principles with their children after the laboratory visit using a scale from 0 = *not at all* to 10 = *a lot*. Parents used the same 11-point scale to rate their rapport with the interventionist, as well as their understanding of, and motivation to learn, the intervention principles.

##### Intervention Adherence

Intervention adherence was assessed by coding parent–child reminiscing narratives that followed the intervention/control instructions and that had been subsequently transcribed verbatim. A previously adapted^17^ coding scheme was used to code for intervention-congruent and incongruent language used by parents and youth (i.e., six codes: words related to positive emotions, negative emotions, anxiety/fear, pain, coping, and bravery). To account for varying narrative lengths, a proportion was calculated for each of the six codes by dividing each by the total number of codes used by each participant. Two researchers blind to group allocation coded a randomly selected 20% (*n* = 4) of the narratives with intercoder reliability ≥.80 (Cohen’s kappa).^32^ The primary coder (T.L.) coded the remaining narratives.

#### Secondary Outcomes

##### Memory Biases

For the purposes of this pilot study, youth memory biases for pain intensity, pain unpleasantness, and pain-related anxiety on day 1 postsurgery and during acute recovery periods (i.e., an average for days 1–3) were secondary outcomes. Pain intensity, unpleasantness, and anxiety were assessed in line with previous research^33^ and to capture both sensory and affective dimensions of the multidimensional pain experience.^34,35^ Memory biases were analyzed and reported to determine the observed effect size of the intervention and to calculate the required sample size for a larger definitive RCT. In line with previous research, memory biases were defined as a within-person deviation between the initial and recalled pain intensity and pain-related unpleasantness/anxiety ratings.^17,20,28^ Negatively biased pain memories were defined as recalling higher levels of pain intensity, unpleasantness, or anxiety compared to initial ratings. Positively biased pain memories were defined as recalling lower levels of pain intensity, unpleasantness, or anxiety than initial ratings.

A trained researcher blind to group allocation conducted an established telephone interview previously used in pediatric surgical cohorts^14,15^ to collect the ratings needed to calculate the memory biases 6 weeks postsurgery. Youth recalled both the sensory (i.e., pain intensity) and affective (i.e., pain unpleasantness and anxiety) aspects of their postsurgical pain at two time points when pain is typically most severe^36^: (1) on day 1 postsurgery and (2) during the acute recovery period (i.e., days 1 to 3 postsurgery); thus, acute recovery encompassed the first time point (i.e., day 1 after surgery). These time points have been used in previous postsurgical pain memory research.^14,33^ Each question was anchored with a specific time frame and location (e.g., day 1 after surgery at the hospital). The same measures (described below) were used in the memory interview as well as the baseline and follow-up questionnaires (i.e., 1 week before surgery, acute recovery [1 to 3 days postsurgery], 2 weeks after surgery, 4 months after surgery).

##### Pain Characteristics and Outcomes

Pain intensity was assessed using an 11-point numeric rating scale (NRS) ranging from 0 (*no pain*) to 10 (*worst pain possible*). The NRS has demonstrated good psychometric properties in pediatric samples undergoing spinal fusion and pectus repair surgeries.^37^

Pain unpleasantness was rated on a 5-point Likert scale assessing how much pain was bothersome over the past 7 days (ranging from 0 = *not at all* to 4 = *very much*). The scale has been previously used in a pediatric perioperative sample.^33^

Pain-related anxiety was assessed using an 11-point NRS (0 = *not anxious/nervous*, 10 = *extremely nervous or anxious*). Similar scales have been used in previous research on children’s pain.^38^

Pain interference was assessed using the pain interference subscale of the PROMIS-25 Profile (Patient-Reported Outcomes Measurement Information System 25-item pediatric short form).^39^ The subscale’s four items are rated on a 5-point Likert scale and assess the extent of everyday impairment due to pain. The scale has excellent psychometric properties and has been used in youth with chronic pain.^40,41^

### Sample Size and Power

The initial RCT was based on a formal sample size estimation (i.e., *n* = 90) that was not appropriate for the present purposes given that the primary outcomes changed when the trial was modified to assess feasibility and acceptability. Samples sizes ranging from 8 to 114 participants are typical for pilot studies examining intervention feasibility and acceptability.^42^ However, we acknowledge that the sample size of 17 parent–child dyads was not initially planned and is the result of the early study termination.

### Statistical Methods

Data analyses were performed using SPSS (v27).^43^ Descriptive statistics were used to characterize the sample and determine intervention feasibility and acceptability. Independent samples *t*, χ^2^, and Fisher’s exact tests compared groups on sociodemographic variables and intervention adherence.

To determine the observed effect size of the intervention on youth pain memories, we conducted six one-way analyses of covariance. In line with previous research,^15,17^ memory biases were defined as a *relative* deviation between the initial and recalled pain ratings. This was statistically modeled by including the initial pain intensity score on day 1 postsurgery as the covariate, memory for pain intensity on day 1 from the 6-week assessment as the dependent variable, and group (intervention or control) as the between-subjects factor.

To calculate the observed effect size of the intervention on youth pain outcomes (i.e., pain intensity and pain interference 4 months postsurgery), we compared the intervention and control groups using a series of independent sample *t* tests.

## Results

**Figure 2.**
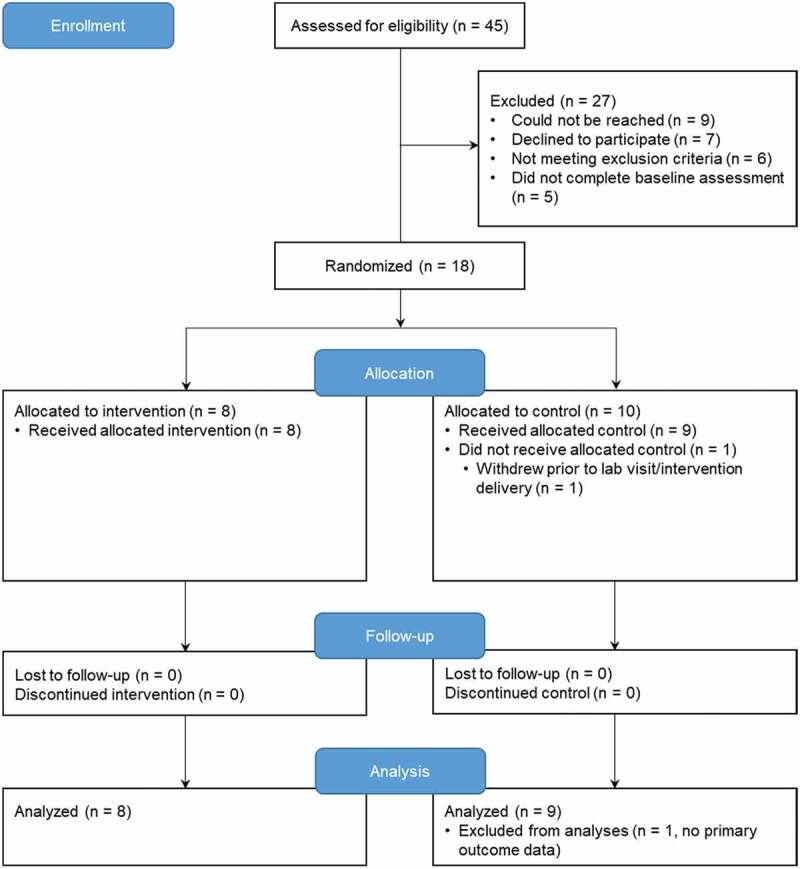
CONSORT flow diagram of trial participants. Enrollment and randomization of participants in the trial.

The RCT was conducted from January 2018 to June 2019; it was stopped due to the lack of funding. Forty-five parent–child dyads were assessed for eligibility (Figure 2). Nine dyads could not be reached prior to surgery; seven families declined to participate. Five dyads did not complete the baseline questionnaires. One dyad did not complete the lab visit and memory interview. There were no significant differences in sociodemographic parameters between participants who completed the study and those who withdrew. Data from 17 parent–child dyads were analyzed. Data were missing at random (Little missing completely at random test *P* = 0.97); no data imputations were performed.

The sample (82% mothers, 76% girls, youth *M*_age_ = 14.1 years, parent *M*_age_ = 49.0 years) was mostly white and educated (73% of parents completed a college degree; Table 1). Most children presented with scoliosis (82%) and underwent spinal fusion (82%). Control and intervention groups did not significantly differ on sociodemographic (Table 1) or initial and recalled levels of pain characteristics (Table 2).

**Table 1.** Demographics and surgery characteristics by group.

Variable	Control group (*n* = 9)	Intervention group (*n* = 8)	Total (*n* = 17)	*P* value^a^
Child age, years, *M* (SD)	14.8 (1.3)	13.4 (2.0)	14.1 (1.8)	0.103
Parent age, years, *M* (SD)	49.4 (5.7)	48.6 (4.2)	49.0 (4.8)	0.767
Child sex, *n* (%)				
Girls	7 (78)	6 (75)	13 (76)	0.893
Boys	2 (22)	2 (25)	4 (24)	
Parent role, *n* (%)				
Mothers	8 (89)	6 (75)	14 (82)	0.453
Fathers	1 (11)	2 (25)	3 (18)	
Parent ethnicity, *n* (%)				
Minority	3 (38)	3 (38)	6 (38)	>0.999
Non-minority	5 (62)	5 (62)	10 (62)	
Child ethnicity, *n* (%)				
Minority	3 (38)	1 (13)	4 (25)	0.248
Non-minority	5 (62)	7 (87)	12 (75)	
Annual household income, *n* (%)				
<$70,000	0 (0)	1 (14)	1 (8)	0.377
≥$70,000	5 (100)	6 (86)	11 (92)	
Parent education, *n* (%)				
High school/vocational school/some college	3 (42)	1 (13)	4 (27)	0.185
College/graduate degree	4 (58)	7 (87)	11 (73)	
Marital status, *n* (%)				
Married/common-law	0 (0)	2 (29)	2 (13)	0.104
Single/divorced	8 (100)	5 (71)	13 (87)	
Children’s presenting diagnosis, *n* (%)				
Scoliosis	7 (78)	7 (88)	14 (82)	0.600
Other	2 (22)	1 (12)	3 (18)	
Surgery type, *n* (%)				
Spinal fusion	7 (78)	7 (88)	14 (82)	0.600
Orthopedic surgery	2 (22)	1 (12)	3 (18)	

The *n*s may not add up to 17 due to missing data.

^a^Independent sample *t* tests, chi-square tests, or Fisher’s exact tests.

**Table 2.** Initial reports of and memory for pain intensity and pain-related fear by group.

Variable	Control group (*n* = 9)	Intervention group (*n* = 8)	Total (*n* = 17)	*P* value^a^
Initial report, *M* (SD)				
Day 1 postsurgery pain intensity, range 3–10	5.8 (1.4)	6.0 (2.5)	5.9 (1.9)	0.805
Day 1 postsurgery pain-related anxiety, range 0–10	3.3 (2.9)	5.8 (2.8)	4.5 (3.1)	0.106
Day 1 postsurgery pain unpleasantness, range 1–4	2.8 (1.0)	2.6 (0.7)	2.7 (0.9)	0.786
Days 1–3 postsurgery pain intensity, range 2.67–9.33	6.0 (1.7)	6.3 (2.2)	6.2 (1.9)	0.770
Days 1–3 postsurgery pain-related anxiety, range 0–9.67	3.5 (3.0)	5.3 (2.5)	4.4 (2.8)	0.242
Days 1–3 postsurgery pain unpleasantness, range 1.33–4	2.6 (1.1)	2.8 (0.7)	2.7 (0.9)	0.643
Pain memories, *M* (SD)				
Memory for day 1 pain intensity, range 1–10	6.9 (2.1)	5.0 (2.8)	5.9 (2.6)	0.150
Memory for day 1 pain-related anxiety, range 0–10	4.7 (3.4)	4.4 (3.0)	4.5 (3.1)	0.909
Memory for day 1 pain unpleasantness, range 1–5	3.3 (0.9)	4.0 (1.4)	3.7 (1.2)	0.253
Memory for days 1–3 pain intensity, range 1–10	6.4 (2.4)	6.3 (3.1)	6.4 (2.6)	0.884
Memory for days 1–3 pain-related anxiety, range 0–10	3.8 (3.0)	4.8 (3.3)	4.2 (3.1)	0.535
Memory for days 1–3 pain unpleasantness, range 1–5	3.0 (1.2)	3.9 (1.5)	3.4 (1.4)	0.199

^a^Independent sample *t* tests.

### Intervention Feasibility

Seventy-four percent (*n* = 17) of enrolled participants completed the study up to the memory interview. All participants (*n* = 8, 100%) allocated to the intervention group received the intervention and competed the study. All but one participants (*n* = 9, 90%) randomized to the control group received attention control instructions and completed the study.

### Intervention Acceptability

Parents reported being motivated to learn the intervention (*M* = 6.9/10, SD 2.4). They understood the purpose of intervention (*M* = 7.8/10, SD 1.9) and reported a good level of rapport with the interventionist (*M* = 7.5/10, SD 1.9). Parents reported using the intervention strategies after the lab visit as 4.4/10 (SD 2.6; 0 = *not at all*, 10 = *a lot*). The intervention was rated as highly acceptable (*M* = 40.8/45, SD 3.9).

### Intervention Adherence

Parents allocated to the intervention group used words associated with memory-reframing principles more frequently compared to participants in the control group (Table 3). Specifically, parents allocated to the intervention group more frequently used words associated with positive emotions, *t*(15) = 2.7, *P* = 0.016, and bravery, *t*(7) = 3.3, *P* = 0.012, and less frequently used words associated with negative emotions, *t*(15) = −2.4, *P* = 0.029, and anxiety/fear, *t*(15) = −2.2, *P* = 0.042, compared to the control group. Parents did not differ in their use of words associated with pain and coping as a function of group allocation (all *P*s > 0.05).

**Table 3.** Narrative codes of parent–child reminiscing about surgery by group.

Narrative codes	Memory-reframing principle	Intervention-congruent action	Control group (*n* = 9), *M* (SD)	Intervention (*n* = 8), *M* (SD)	Total (*n* = 17)	*P* value^a^
Parent use						
Positive emotion	(1) Focusing on positive aspects of pain experience	Use more	0.2 (0.2)	0.4 (0.1)	0.3 (0.2)	0.016
Negative emotion	(1) Focusing on positive aspects of pain experience	Use less	0.3 (0.1)	0.1 (0.1)	0.2 (0.1)	0.029
Anxiety/fear	(2) Decreasing exaggerations in memories for pain/fear	Use less	0.2 (0.1)	0.1 (0.1)	0.1 (0.1)	0.042
Pain	(2) Decreasing exaggerations in memories for pain/fear	Use less	0.3 (0.1)	0.2 (0.2)	0.2 (0.2)	0.727
Coping	(3) Building up pain-specific pain efficacy	Use more	0.1 (0.1)	0.1 (0.1)	0.1 (0.1)	0.871
Bravery	(3) Building up pain-specific pain efficacy	Use more	<0.1 (0.1)	0.1 (0.1)	<0.1 (<0.1)	0.012
Youth use						
Positive emotion	(1) Focusing on positive aspects of pain experience	Use more	0.1 (0.2)	0.3 (0.2)	0.2 (0.2)	0.055
Negative emotion	(1) Focusing on positive aspects of pain experience	Use less	0.2 (0.2)	0.1 (0.1)	0.2 (0.2)	0.130
Anxiety/fear	(2) Decreasing exaggerations in memories for pain/fear	Use less	0.2 (0.1)	<0.1 (0.1)	0.1 (0.1)	0.045
Pain	(2) Decreasing exaggerations in memories for pain/fear	Use less	0.5 (0.3)	0.3 (0.2)	0.4 (0.3)	0.266
Coping	(3) Building up pain-specific pain efficacy	Use more	<0.1 (0.1)	0.1 (0.1)	0.1(0.1)	0.337
Bravery	(3) Building up pain-specific pain efficacy	Use more	0	0	0	—

The values represent the proportion of words associated with each content theme over the total number of codes used.

^a^Independent sample *t* tests.

Youth use of content codes did not significantly differ across two groups except for anxiety-/fear-related words (*t*(11) = −2.3, *P* = .045) that were used less frequently by youth in the intervention group compared to the control group.

### The Effect of Intervention on Youth Pain Memories

At the 6-week follow-up, groups did not differ on memory biases for day 1 or acute recovery pain intensity, anxiety, or pain unpleasantness (all *P*s > 0.05; Table 4). The largest effect size for the intervention was observed for youth memory for day 1 pain intensity (*η_p_*^2^ = .22, *P* = 0.074) with youth allocated to the intervention group (*M* = 4.9/10, SD 2.8, 95% confidence interval [CI] 3.3–6.5) recalling pain in a more accurate or positively biased way compared to the control group (*M* = 6.9/10, SD 2.1, 95% CI 5.4–8.6). To detect an *η_p_*^2^ = 0.22 effect size with a type I error of 0.05, 80% probability, and two covariates (i.e., age and gender), a sample of 203 youth would be required.

**Table 4.** Youth memory biases as a function of group allocation.

	Group (adjusted mean, 95% CI)^a^					
Criterion	Control	Intervention	Mean differences^b^ (95% CI)	*F* value	*P* value^c^	*η_p_^2^*
Memory for pain intensity (day 1) (*n* = 16)	6.6 (5.4–8.6)	4.9 (3.3–6.5)	2.1 (−0.2 to 4.3)	3.76	0.074	0.224
Memory for pain unpleasantness (day 1) (*n* = 16)	3.1 (2.3–3.9)	4.0 (3.2–4.9)	−0.9 (−2.1 to 0.3)	2.69	0.125	0.171
Memory for pain-related anxiety (day 1) (*n* = 16)	4.6 (2.6–6.6)	3.6 (1.7–5.6)	1.0 (−1.9 to 3.9)	0.54	0.476	0.040
Memory for pain intensity (days 1–3) (*n* = 16)	6.2 (5.1–7.2)	6.1 (5.0–7.1)	0.1 (−1.4 to 1.6)	0.02	0.904	0.001
Memory for pain unpleasantness (days 1–3) (*n* = 16)	2.8 (2.0–3.7)	3.8 (2.9–4.6)	−1.0 (−2.2 to 0.2)	2.98	0.108	0.186
Memory for pain-related anxiety (days 1–3) (*n* = 16)	4.7 (3.6–5.8)	3.8 (2.7–4.9)	0.8 (−0.8 to 2.4)	1.19	0.295	0.084

^a^The adjusted means are derived from the analyses of covariance controlling for corresponding initial pain and pain-related fear ratings, as well as child sex.

^b^Control minus intervention group scores.

^c^Analyses of covariance.

### The Effect of Intervention on Youth Pain Outcomes

Intervention and control groups did not significantly differ on pain intensity or interference at 4-month follow-up (all *P*s > 0.05). The largest effect size of the intervention was observed for youth pain interference (*η_p_*^2^ = 0.23), such that youth in the intervention group reported lower levels of pain interference (*M* = 48.3, SD 4.5) than youth in the control group (*M* = 52.6, SD 4.5). To detect an *η_p_*^2^ = 0.22 effect size with a type I error of 0.05, 80% probability, and two covariates (i.e., age and gender), a sample of 186 youth would be required.

## Discussion

The goal of this pilot RCT was to assess feasibility and acceptability of a memory-reframing intervention in a sample of youth undergoing major surgery. The study also aimed to assess participants’ adherence to the intervention principles. Recruitment and parent report indicated good feasibility.^30^ Parents allocated to the intervention group rated the intervention as highly acceptable. The feasibility and acceptability ratings of the intervention are in line with previously reported ratings of a similar intervention tested in a sample of young children undergoing minor surgery.^17^ Parents in the intervention group followed the intervention principles when reminiscing with their children about their recent surgery. In contrast, youth allocated to the intervention group did not follow intervention instructions. There were no statistically significant differences in youth pain memories or pain outcomes as a function of group membership. Nevertheless, the observed effect sizes provide an estimate of the required sample size for a definitive RCT testing the efficacy of the intervention.

Intervention adherence varied among parents and youth. Similar to the previous study with young children undergoing tonsillectomy,^17^ parents randomized into the intervention group used words congruent with the intervention principles (i.e., using words associated with positive emotions and bravery more frequently; using words associated with negative emotions and anxiety/fear less frequently). However, there were no significant differences between the groups in parent use of words associated with pain and coping strategies. Previous research^28^ has demonstrated the role of parent reminiscing content in the development of children’s memories for pain. More frequent parent use of pain-related words was associated with more negatively biased memories for postsurgical pain in young children.^28^ Further, when parents attend more to pain immediately before or during acute pain experiences (e.g., needle procedures), children are observed to experience more distress and pain.^44^ High frequencies of pain-related words when reminiscing about past painful experiences may increase children’s distress and bring the distressing sensory aspects of past pain into focus. Thus, it would be important to further emphasize the importance of and model avoiding pain-laden language. Reminding children about successful coping skills is another key part of pain memory reframing.^23^ It may be challenging for parents to recall coping strategies that worked for their children due to their own distress,^45^ which may explain nonsignificant differences in use of coping language across the two groups. The intervention may be adjusted to more explicitly encourage parents and youth to recall successful coping skills. The recalled coping skills may, then, be incorporated into visual reminders to be used during and after the intervention, similar to Chen and colleagues’ memory-reframing intervention.^23^

In the present study, youth were present for the intervention/control instructions. Therefore, youth use of intervention-congruent and incongruent words was examined in addition to parent intervention adherence. Youth did not use any bravery-related words when reminiscing about their surgery with parents. Youth also did not differ in their use of words associated with positive/negative emotions, pain, and coping. However, youth in the intervention group used fear-/anxiety-related words less frequently compared to the control group. Previous observational research on child reminiscing patterns demonstrated that young children who used more emotion-laden language when reminiscing about past tonsillectomy had more positively biased or accurate pain memories.^28^ The use of words associated with negative emotions while reminiscing may, however, depend on the levels of experienced stress. In a study of children aged 2 to 13 years who suffered a stressful injury, went to an emergency department for treatment, and recalled it later, the use of emotion-laden language differed.^46^ Children who, according to their parents’ ratings, found their experience to be highly distressing provided less details about any of their emotions compared to children who were less distressed.^46^ The intervention instructions therefore may be tailored to children’s levels of distress associated with their postsurgical experience. Distressed youth may need more reminders and encouragement to introduce positive emotion–related words into their reminiscing narratives. Negative emotion–focused words may need to be further discouraged. In a sample of adolescents who reminisced with their caregivers about a traumatic natural disaster event (i.e., a tornado), the more frequent mentions of negative emotion words by adolescents were associated with higher levels of adolescents’ anxiety.^47^ Children may naturally use negative emotion words less frequently when reminiscing about past events involving pain compared to past events involving sadness.^48^

The intervention did not significantly change youth memories for pain, nor did it influence youth pain outcomes 4 months postsurgery. These null results are likely due to the small, underpowered sample size of the present pilot study. Preliminary analyses were performed to calculate the sample size calculation required for a larger RCT examining the intervention efficacy in youth undergoing major surgery. The larger RCT’s preregistered sample size of 90 youth was based on medium effect sizes observed in previous memory-reframing interventions.^24^ However, a recently published RCT of the intervention efficacy in young children revealed a smaller effect size of the intervention on children’s memories for pain.^17^ Based on the effect size observed in the current study, a sample size of at least 203 youth would be required to detect the effect of intervention on youth pain memories. Further, the potential effect size of the intervention on youth pain outcomes was hypothesized based on the memory-reframing interventions that were tested in procedural pain contexts.^21,23^ To our knowledge, no studies tested the efficacy of memory reframing in the context of postsurgical pain. Preliminary analyses were needed to examine the observed effect size and adjust sample calculations. The observed effect was largest for pain interference at 4 months postsurgery. To detect an effect of a similar size, a sample of 186 youth would be required. Based on these preliminary analyses, the registered sample size of the larger RCT (NCT03110367) will be changed to 250 parent–child dyads with a 20% overrecruitment to account for attrition.

The intervention acceptability was measured using parent report only, which does not align with the values of and initiatives for patient engagement in study design and development. Nevertheless, after the study was completed, a youth patient partner (J.S.) provided her feedback on the intervention as well as ways to improve it.

Based on patient partner feedback, the following modifications of the intervention should be considered in the future trials. First, more active involvement of youth participants in the intervention design and the intervention procedure should be incorporated. Given adolescents’ increasing independence and cognitive capacity, playing a more active role in treatment, as well as being given voice and space to share, construct, and reconstruct their experience of pain, is developmentally appropriate and in line with treatment benefits identified by youth with chronic pain.^49^ Second, providing a more in-depth rationale for the importance of pain memories before the surgery and/or including the elements of intervention throughout the surgery preparation period would allow youth to better understand and get more invested in the intervention. Third, according to the patient partner, the intervention could be improved by explicitly validating and supporting youth during reminiscing. Indeed, interpersonal validation in pain communication was shown to be an important factor influencing affect and report of pain intensity in patients with chronic pain.^50^ In conversations about chronic pain, validation may convey the listener’s acceptance, understanding, and confirmation that another’s pain is complex, distressing, and legitimate. In a study of interpersonal validation and empathy in adults with chronic pain and their partners, higher levels of validation were linked to higher levels of disclosure about pain experiences.^51^ No studies have examined the levels of validation and empathic support in parent–child conversations about pain. The patient partner also highlighted the importance of asking youth postintervention whether they thought that reminiscing about their past pain was helpful and whether their parent was supportive/validating and focused on positive aspects of their past pain experience.

There are limitations to this study. First, the original RCT was discontinued before the primary goals (i.e., examining the efficacy of the intervention to change youth pain memories and improve pain outcomes) were achieved. However, the importance of publishing reports of clinical trials that were discontinued and/or demonstrated null results has been emphasized.^52^ Further, this study’s data and preliminary results will be beneficial for future trials of the intervention. Second, the intervention acceptability assessment was limited to parent report, as well as quantitative methods. In a future RCT we are planning, both parents and youth will be invited to provide their qualitative feedback regarding the intervention. Third, the intervention was designed to take place in person. The ongoing COVID-19 pandemic highlighted the urgent need for virtually delivered interventions. Given the flexible format of the memory-reframing intervention, it can be adapted to be delivered online and practiced at home. The virtual delivery would have an advantage of greater inclusivity, because families would not need to spend time and money traveling to the laboratory visit. Further, parent–child dyads would be learning and applying the intervention principles in their usual home environment, which may increase the use of intervention principles by forming context-dependent (i.e., home) memories.^53^ Thus, simply being in the home environment where participants received the intervention would remind them about pain memory reframing. Fourth, the intervention reminders were limited to one handout summarizing the intervention principles. Additional, visually attractive reminders (as used by Chen and colleagues^23^) may be more effective in capturing parent and youth attention and encouraging them to use the intervention principles more frequently. The intervention was limited to a single occurrence to reduce burden on families and increase intervention feasibility. However, repeated, versus one-time, memory-reframing instances may be more efficacious in changing memories for past event.^54^ Future trials should consider repeated encouragement to reminisce about past pain using the intervention principles, which may be achieved using text/e-mail reminders. We have also recently argued for the inclusion of memory-reframing principles into preparation for painful procedures.^55^ An abbreviated version of the intervention principles with the focus on building up pain-related self-efficacy and reminding youth about past successful coping strategies may be included in future trials in preparation for surgery. Finally, it was not possible to blind participants and the interventionist to group allocation, which is common for psychosocial interventions.^56^

In conclusion, this pilot trial examined the feasibility and acceptability of, as well as adherence to, a memory-reframing intervention in a sample of youth undergoing major surgery. The intervention was feasible. Parents reported it to be highly acceptable. Parents, but not youth, adhered to its principles when reminiscing about past surgery. The preliminary analyses did not reveal significant effects of the intervention on youth pain memories or pain outcomes. The observed effect sizes were used to inform the sample size of a larger RCT.

## Supplementary Material

Supplemental Material
